# Objective estimation of colonic transit time using radiopaque markers in an abdominal X-ray after laparoscopic colorectal resection: secondary analysis of a randomized clinical trial

**DOI:** 10.1093/bjsopen/zrad111

**Published:** 2023-11-01

**Authors:** Yanic Ammann, Rene Warschkow, Stephan Bischofberger, Kristjan Ukegjini, Ignazio Tarantino, Thomas Steffen

**Affiliations:** Department of General, Visceral, Endocrine, and Transplant Surgery, Cantonal Hospital of St Gallen, St Gallen, Switzerland; Department of General, Visceral, Endocrine, and Transplant Surgery, Cantonal Hospital of St Gallen, St Gallen, Switzerland; Department of General, Visceral, Endocrine, and Transplant Surgery, Cantonal Hospital of St Gallen, St Gallen, Switzerland; Department of General, Visceral, Endocrine, and Transplant Surgery, Cantonal Hospital of St Gallen, St Gallen, Switzerland; Department of General, Visceral, Endocrine, and Transplant Surgery, Cantonal Hospital of St Gallen, St Gallen, Switzerland; Department of General, Visceral, Endocrine, and Transplant Surgery, Cantonal Hospital of St Gallen, St Gallen, Switzerland

## Introduction

Postoperative ileus has become a public health problem due to its role in postoperative morbidity, prolonged hospital stays, and increased costs^1–3^. This condition occurs at a rate of between 15 and 20 per cent after colorectal resection^4^. The Enhanced Recovery After Surgery (‘ERAS’) group recommended a multimodal approach in the prophylaxis and treatment of postoperative ileus after colorectal resection^5^. The impact of factors such as sex, age, and BMI on postoperative ileus remains unclear. Postoperative ileus is commonly determined by time to first flatus, time to first defaecation, and time to first tolerance of solid food intake^6–10^. The diagnostic value of colonic transit time (CTT) in comparison with postoperative ileus and other measurements is unknown. A promising technique to estimate CTT is the ingestion of radiopaque markers, followed by an abdominal X-ray^11,12^. After ingestion, the radiopaque markers pass through the gastrointestinal tract and allow intestinal transit to be timed. An increase in postoperative CTT could be a sign of postoperative ileus.

The aim of this retrospective secondary analysis of the previously published double-blinded, placebo-controlled, randomized CaCo trial^13^ was to compare the diagnostic reliability of alternative measures of postoperative ileus (time to first flatus, time to first defaecation, and time to first tolerance of solid food intake) with prolonged CTT after elective laparoscopic colorectal surgery.

## Methods

### Registration

The trial was registered with ClinicalTrials.gov (NCT02510911) and the Swiss National Clinical Trials Portal (SNCTP000001131) for the primary analysis.

### Ethics approval

The previously published study protocol of the primary analysis was approved by the Ethics Committee of the Canton St Gallen (EKSG 15/023) and Swissmedic (Swiss Agency for Therapeutic Products; www.swissmedic.ch) before any patients were enrolled in this trial.

### Informed consent

Written consent by the study participants was provided before enrolment in the study.

### Setting and participants

Eligible patients were aged greater than or equal to 18 years and were undergoing elective laparoscopic colon or upper rectum resection without a significant change in surgery (no conversion to open, stoma formation, additional resection of adjacent organs, or other major unplanned procedures) or the use of epidural anaesthesia during or after surgery, with full availability of the radiopaque marker data for all 3 days. See *Text S1* for further information about the exclusion criteria and baseline study participant characteristics.

Postoperative ileus was defined as a delay in the return of bowel function requiring nasogastric tube insertion, repeated vomiting, or intolerance of solid food intake after postoperative day (POD) 5.

Time to first tolerance of solid food intake was measured from the end of surgery until the patient tolerated the intake of solid food (any food requiring chewing). Tolerance of food was defined as the first time that a patient could eat without vomiting or experiencing significant nausea within 4 h after a meal and without returning to only receiving oral fluids. See *Text S1* for further information.

### Intervention

The study participants ingested a capsule containing 10 radiopaque markers (Colon Transit Radiopaque Markers (P&A Mauch, Münchenstein, Switzerland), Ref: CTT6V10, CE1253) every morning at breakfast on POD 1–3. The markers had a different geometric form each day and 3 × 10 = 30 markers were ingested in total throughout the study. On POD 4, one single abdominal X-ray imaging procedure (posterior–anterior) was performed to determine the locations of the radiopaque markers. The position (left colon, right colon, rectosigmoid, and excreted positions) and number of the radiopaque marker was determined using the method described by Metcalf *et al*.^11^. See *Text S1* and *Fig. S1* for further information.

### Outcome

The primary outcome was to compare the diagnostic reliability of alternative measures of postoperative ileus (time to first flatus, time to first defaecation, and time to first tolerance of solid food intake) with prolonged CTT. See *Text S1* for the secondary outcomes.

### Statistical analysis

Statistical analyses were performed using R statistical software (www.r-project.org). See *Text S1* for detailed information.

## Results

Of the 60 patients included in the CaCo trial^13^, nine patients were excluded because of missing or incomplete radiopaque marker data. See *Text S1* and *Tables S1 and S2* for more information about radiopaque marker distribution.

The characteristics of the study participants were compared at the median dichotomized CTT in participants with a CTT of less than 44 h (short CTT) and a CTT of greater than or equal to 44 h (long CTT) (*Table 1*). The time to first tolerance of solid food intake was significantly faster for patients with a short CTT compared with patients with a long CTT (50.3 ± 19.6 *versus* 69.3 ± 26.7 h respectively; *P* = 0.022). In both groups (short CTT *versus* long CTT), time to first flatus (35.2 ± 13.4 *versus* 45.4 ± 23.2 h respectively; *P* = 0.140) and time to first defaecation (66.0 ± 24.5 *versus* 67.7 ± 28.2 h respectively; *P* = 0.933) were similar. Postoperative ileus (only repeated vomiting) was observed between POD 1 and 6 in 11 patients (44.0 per cent) with a long CTT and in four patients (15.4 per cent) with a short CTT (*P* = 0.023).

**Table 1:** Baseline characteristics and postoperative data for short colonic transit time and long colonic transit time divided at the median dichotomized colonic transit time of 44 h

Variable	Total; *n* = 51	Short CTT (<44 h); *n* = 26	Long CTT (≥44 h); *n* = 25	*P*
**CTT (h)**				
Mean (s.d.)	41.1 (21.8)	23.4 (13.9)	59.4 (10.2)	<0.001*†
Median (i.q.r.)	43.2 (24.0–58.8)	24.0 (10.2–36.0)	60.0 (48.0–67.2)	
**Sex**				
Male	30 (58.8)	17 (65.4)	13 (52.0)	0.332‡
Female	21 (41.2)	9 (34.6)	12 (48.0)	
**Age (years)**				
Mean (s.d.)	62.9 (9.7)	62.5 (10.4)	63.3 (9.2)	0.874*
Median (i.q.r.)	63.2 (56.7–70.1)	66.4 (53.2–70.3)	62.1 (56.8–69.2)	
<65	27 (52.9)	12 (46.2)	15 (60.0)	0.322‡
≥65	24 (47.1)	14 (53.8)	10 (40.0)	
**BMI (kg/m^2^)**				
Mean (s.d.)	26.4 (4.5)	26.5 (4.0)	26.3 (5.1)	0.620*
Median (i.q.r.)	26.2 (23.4–29.1)	26.5 (24.2–28.3)	24.8 (22.5–29.4)	
<30	41 (80.4)	22 (84.6)	19 (76.0)	0.467§
≥30	10 (19.6)	4 (15.4)	6 (24.0)	
**Height (cm)**				
Mean (s.d.)	169.6 (9.3)	172.4 (9.1)	166.7 (8.8)	0.030*†
Median (i.q.r.)	170.0 (161.0–177.0)	175.5 (165.2–179.5)	167.0 (160.0–173.0)	
**Weight (kg)**				
Mean (s.d.)	75.8 (13.3)	78.9 (12.9)	72.6 (13.2)	0.088*
Median (i.q.r.)	74.0 (66.5–87.0)	83.0 (68.5–89.2)	70.0 (66.0–81.0)	
**Type of surgery**				
Sigmoid resection	39 (76.5)	19 (73.1)	20 (80.0)	0.184¶
Right hemicolectomy	5 (9.8)	3 (11.5)	2 (8.0)	
Low anterior resection	3 (5.9)	3 (11.5)	0 (0.0)	
Segmental resection	2 (3.9)	0 (0.0)	2 (8.0)	
Left hemicolectomy	1 (2.0)	0 (0.0)	1 (4.0)	
High anterior resection	1 (2.0)	1 (3.8)	0 (0.0)	
**Side of operation**				
Left	45 (88.2)	23 (88.5)	22 (88.0)	0.962§
Right	6 (11.8)	3 (11.5)	3 (12.0)	
**Operating time (min)**				
Mean (s.d.)	171.4 (51.6)	163.4 (55.8)	179.8 (46.5)	0.127*
Median (i.q.r.)	159.0 (134.5–204.5)	152.0 (126.5–181.3)	180.0 (149.0–213.0)	
**Clavien–Dindo classification**				
0	39 (76.5)	20 (76.9)	19 (76.0)	1.000¶
I	2 (3.9)	1 (3.8)	1 (4.0)	
II	7 (13.7)	4 (15.4)	3 (12.0)	
IIIb	2 (3.9)	1 (3.8)	1 (4.0)	
IVa	1 (2.0)	0 (0.0)	1 (4.0)	
**Morphine use**				
No	31 (60.8)	18 (69.2)	13 (52.0)	0.208‡
Yes	20 (39.2)	8 (30.8)	12 (48.0)	
**Postoperative ileus**				
No	36 (70.6)	22 (84.6)	14 (56.0)	0.025†‡
Yes	15 (29.4)	4 (15.4)	11 (44.0)	
Intolerance of solid food intake after POD 5	0 (0.0)	0 (0.0)	0 (0.0)	
Repeated vomiting	15 (29.4)	4 (15.4)	11 (44.0)	
Nasogastric tube insertion	0 (0.0)	0 (0.0)	0 (0.0)	
**Time to first flatus (h)**				
Mean (s.d.)	40.2 (19.3)	35.2 (13.4)	45.4 (23.2)	0.140*
Median (i.q.r.)	37.6 (25.6–48.2)	31.0 (25.6–44.8)	41.0 (28.9–52.8)	
**Time to first defaecation (h)**				
Mean (s.d.)	66.9 (26.1)	66.0 (24.5)	67.7 (28.2)	0.933*
Median (i.q.r.)	62.4 (49.2–78.5)	62.4 (51.4–76.9)	62.4 (48.8–93.1)	
**Time to first tolerance of solid food intake (h)**				
Mean (s.d.)	59.6 (25.0)	50.3 (19.6)	69.3 (26.7)	0.022*†
Median (i.q.r.)	48.5 (44.0 to 73.0)	47.5 (43.0–53.4)	68.8 (45.6–86.9)	
**Time to discharge from hospital (days)**				
Mean (s.d.)	6.5 (2.9)	6.0 (2.9)	7.1 (3.0)	0.070*
Median (i.q.r.)	5.8 (5.0–7.0)	5.1 (4.9–6.1)	6.1 (5.1–7.2)	

Values are *n* (%) unless otherwise stated. *Mann–Whitney *U* test. †Significant. ‡Chi-squared test. §Mid-*P* test. ¶Chi-squared test, Monte Carlo simulated. CTT, colonic transit time; i.q.r., interquartile range; POD, postoperative day.

**Fig. 1:**
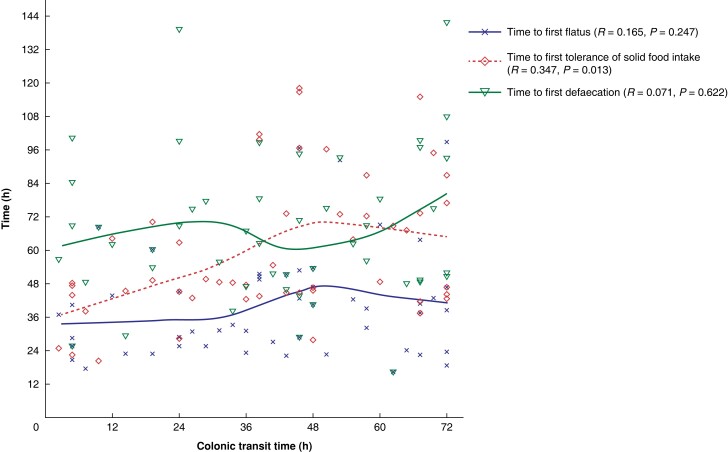
Correlation between colonic transit time and time to first flatus, time to first defaecation, and time to first tolerance of solid food intake

The time to first tolerance of solid food intake was positively correlated with CTT (*R* = 0.347, *P* = 0.013); however, CTT was not correlated with time to first flatus (*R* = 0.165, *P* = 0.247) or time to first defaecation (*R* = 0.071, *P* = 0.622) (*Fig. 1*). See *Text S1* for more correlation information.

## Discussion

Current data regarding radiopaque marker intake and distribution are rare. This study provides a descriptive and illustrative analysis of radiopaque marker distribution in patients who had undergone elective laparoscopic colorectal resection. Among other commonly used measures of postoperative ileus, time to first tolerance of solid food intake was positively correlated with longer CTT. There were no such correlations for the time to first flatus and time to first defaecation. These results raise questions regarding their wide usage in studies for the assessment of postoperative ileus.

Early feeding is thought to improve gastrointestinal motility and passage, as well as postoperative ileus^5,14–16^. This analysis describes a significant positive correlation between time to first tolerance of solid food intake and CTT, and thus a significant diagnostic value of a long CTT in the prediction of the time to first tolerance of solid food intake. As correlation does not indicate causality, distinguishing between cause or effect is difficult. It has to be questioned whether fast colonic passage leads to an early start of intake of solid food or vice versa.

Time to first flatus and time to first defaecation do not seem to be reliable measures of CTT. The frequency, consistency, quantity, and urgency of bowel movements exhibit considerable variability^17^. The detection of bowel movements is subjective and patient associated. It can be challenging to measure in patients who have been recently operated on, especially during the night and during physical activity. Discussions about personal topics such as flatus or defaecation can be difficult for some patients^18^. As the CaCo trial was powered for time to first defaecation, the data regarding the positions of the radiopaque markers in this study can allow for better sample size calculations in further follow-up studies^13^.

The time to discharge from hospital measures the consequence of postoperative ileus. It depends on a variety of medical, administrative, and financial factors, in addition to gastrointestinal passage. An impaired general condition or the occurrence of postoperative complications can prolong the duration of a hospital stay. The lack of an appropriate rehabilitation unit or a suitable nursing facility can also result in a prolonged hospital stay. Healthcare systems with tariff systems (including the case flat rate) set incentives for the optimal length of hospital stay. Furthermore, the wishes of a patient and their relatives regarding the optimal time of discharge can vary significantly. This scenario is often corrected by differentiation of the medically indicated *versus* the actual length of hospital stay.

X-ray radiopaque markers and the time to first tolerance of solid food intake are valid ways to assess colonic transit and postoperative ileus after elective laparoscopic colorectal resection.

## Supplementary Material

zrad111_Supplementary_Data

## Data Availability

The data that support the findings of this study are available in an anonymous form from the corresponding author upon reasonable request.
