# Supplementary material for: Objective estimation of colonic transit time using radiopaque markers in an abdominal X-ray after laparoscopic colorectal resection: secondary analysis of a randomized clinical trial
Source: BJS Open. 2023 Nov 1;7(6):zrad111. doi: 10.1093/bjsopen/zrad111 (PMC10627524; doi:10.1093/bjsopen/zrad111)

**Objective estimation of colonic transit time by radiopaque markers in an abdominal X-ray after laparoscopic colorectal resection: A** **secondary analysis of a randomized controlled trial**

Yanic Ammann^1^; Rene Warschkow^1^; Stephan Bischofberger^1^; Kristjan Ukegjini^1^; Ignazio Tarantino^1^; Thomas Steffen^1^

^1^ Department of General, Visceral, Endocrine, and Transplant Surgery, Cantonal Hospital of St. Gallen, St. Gallen, Switzerland

**Corresponding author**

Thomas Steffen, Department of General, Visceral, Endocrine, and Transplant Surgery, Cantonal Hospital of St. Gallen, Rorschacherstrasse 95, CH-9007 St. Gallen, Switzerland, e-mail: [thomas.steffen@kssg.ch](mailto:thomas.steffen@kssg.ch), ORCID iD 0000-0001-9374-5703

**Supplementary Materials – Index**

**Supplementary Text, Figures and Tables**

**Supplementary Text S1** page 2 - 13

**Table S1 –** Radiopaque marker indigested on postoperative day (POD) page 14
1, 2, and 3 and 1-3. The position was obtained from an abdominal X-ray
on POD 4.

**Table S2 –** Uni- and multivariable mixed effects cumulative link model page 14
for ordinal distribution of radiopaque markers on X-ray.

**Figure S1 –** Abdominal X-ray imaging (posterior-anterior) with division page 15
of the large intestine in the right colon (R), left colon (L), and
rectosigmoid (RS) according to the method described by Metcalf et al. (9)

**Supplementary Text S1**

**Methods**

*Setting and participants*

This study used a secondary retrospective analysis of the previously published double-blinded, placebo-controlled, randomized CaCo trial with three arms (1). Dosages of 300 mg and 600 mg caffeine were not superior to placebo in terms of time to first bowel movement, flatus, solid food intake, discharge from hospital, and CTT. The primary endpoint of the CaCo trial was: time to the first bowel movement measured from the time of wound closure to the patient’s first bowel movement in hours by a study nurse (1, 2).

The exclusion criteria of the CaCo-trial included ASA-Score IV or V, previous extensive abdominal surgery, inflammatory bowel disease, reoperation before the return of normal bowel function, and other factors (2).

Baseline study participant characteristics included sex, age, body mass index (BMI), height, weight, type of surgery, duration of surgery, postoperative morbidity (Clavien‒Dindo Classification (3)) morphine use, time to first flatus, time to first defecation, time to tolerance of first solid food intake, and time to discharge from hospital.

*Intervention*

Preoperative bowel preparation was handled differently according to the type of surgery. Right sided colon resection did not receive any preoperative bowel preparation. Left sided colon resection receive preoperative bowel preparation with an anal enema. In anterior / higher rectum resection preoperative bowel preparation was performed routinely with two liters of oral laxative (Moviprep®, Norgine AG, 6005 Luzern, Switzerland, registration number Swissmedic: 57900).

If a nasogastric tube was in place, it was pinched off for 30 minutes after intake of the radiopaque markers or of any medication. The sack and in case of emesis also the vomit was checked regularly by a nurse regarding the radiopaque markers. If one or more radiopaque markers were not ingested correctly, the patient was excluded from the analysis.

Postoperative management was carried out according to the clinic-internal standard, which is derived from the guidelines of the ERAS group recommendation (4). There was no difference for right-sided, left-sided or anterior / higher rectum resection. Mild laxative medication was prescribed regularly for all patients starting on postoperative day 1. This include Importal® (Zambon Schweiz AG, 6814 Cadempino, Switzerland, registration number Swissmedic: 52785 and 46787 Swissmedic), Laxoberon® (Opella Healthcare Switzerland AG, Risch, Switzerland, registration number Swissmedic: 37871), and Magnesia San Pellegrino® (Tentan AG, 4452 Itingen, Switzerland, registration number Swissmedic: 10574 and 27481).

The decision if a patient was presented solid food was done interdisciplinary by the ward doctor, the surgeon, and the nurse. The prerequisite was the tolerance of a fluid / non-chewing diet. The patient was able to take fluids at any time during day and night. Regular solid food was served at 7 am, 12 noon and 18 pm. Between these times, the patients were able to order rusk, rice cake, and crispbread. The decision to serve solid food could be made at any time.

*Outcome*

Secondary outcomes:

- Distribution of the radiopaque markers on POD 4.
- Influencing factors (morphine use, sex, age, BMI, and side of operation) on CTT.
- Influencing factors on colonic transit regarding the ordinal distribution of the radiopaque markers by using the modern statistical analysis method of (CLMMs).

*Statistical analysis*

The data were prospectively collected by a dedicated study nurse from the study participants via direct collection, the medical records, and a questionnaire administered before and during hospitalization. The data were entered into a custom-made, scientific, and relational software database with extensive data entry checks. This analysis was performed retrospectively.

Baseline study participant characteristics were median dichotomized in two groups regarding the calculated CTT. A two-sided *P value* of less than 0.05 was considered to be statistically significant. Continuous data were expressed as the mean ± standard deviation (SD) or the median with interquartile range (IQR). Chi-squared statistics were used to compare proportions, and Mann‒Whitney U and Kruskal‒Wallis tests were used to compare the continuous variables. The association of continuous data was assessed by nonparametric correlation analysis using the spearman rank correlation coefficient. For the regression analysis, *p values* were estimated via likelihood-ratio tests. The Wald method obtained 95% confidence intervals (95% CIs) of continuous values and for categorical data by Agresti and Coull (5). To compare other measures of postoperative ileus, the median dichotomized CTT was defined as the gold standard. The diagnostic value of the different measures was assessed as the continuous variables. First, receiver operating curves (ROCs) were estimated with the trapezoidal rule, and the area under the ROC curve (AUC) was calculated with its 95% CI based on 1,000 simulations. The optimal cut-off for the measures was estimated by using a kernel-based function from the “cutpointr” R library (6). Based on these cut-off values, 2-by-2 tables with true and false-positives and negatives were obtained to estimate the diagnostic value in terms of sensitivity, specificity, and overall predictive accuracy with their 95% CIs. The ordinal distribution of the radiopaque marker position on the X-ray on POD 4 was analysed by fitting CLMMs. In this model, the ordinal distributed marker position (right side < left side < rectosigmoid < excreted) is interpreted as categories of a latent variable (7). The models were estimated with the adaptive Gauss-Hermite quadrature approximation with 30 quadrature points with the day of indigestion of the marker as a random intercept and with flexible thresholds by using the R library ordinal. To avoid overfitting, radiopaque markers that were found in the small bowel had to be aggregated with the right colon.

**Results**

All 1,530 radiopaque markers had passed the stomach when the X-ray was performed. Of the 1,530 markers, 253 indigested on POD 1 (16.5%), 240 (15.7%) indigested on POD 2, and 164 (10.7%) indigested on POD 3 were excreted (Table S1). The mean CTT was 41.1±21.8 h, and the median CTT was 43.2 h (IQR: 24.0-58.8 h).

Corresponding to the analysis of correlation, time to tolerance of first solid food intake predicted a long CTT of 44 h or more (AUC=0.69, *P*=0.023), with an optimal cut-off value for time to first solid food intake of 59.5 h (95% CI: 53.5-76.4 h). The overall predictive accuracy was 67% (95% CI: 67-80%), with a sensitivity of 59% (95% CI: 41-77%) and a specificity of 73% (95% CI: 58-89%). No significant diagnostic value in the prediction of a long CTT of 44 h or more was observed for time to first flatus (AUC=0.62, *P*=0.139), and defecation (AUC=0.51, *P*=0.932) with predictive accuracies of 64% (95% CI: 51-76%), and 55% (95% CI: 41-68%) respectively.

When considering the ordinal distribution of the position of the markers in CLMMs, less advanced positions of the radiopaque marker were observed in patients who were treated with morphine (OR=0.44, 95% CI: 0.32-0.59, *P<*0.001) and in female patients (OR=0.44, 95% CI: 0.33-0.60, *P<*0.001). A more advanced position of the markers was observed after right colorectal resection (OR=2.02, 95% CI: 1.21-3.38, *P*=0.006). No significant effect was observed for age, BMI, and caffeine compared to the placebo after multivariable adjustment (Table S2).

No difference in mean CTT was observed between the three arms of the CaCo trial (placebo: 46.3±20.6 h vs. 300 mg caffeine: 34.2±22.9 h vs. 600 mg caffeine: 41.8±21.7 h; p=0.317), male vs. female patients (38.6±19.2 h vs. 44.6±25.1 h, respectively; p=0.275), left vs. right colorectal resection (41.1±21.4 h vs. 40.9±26.6 h, respectively; p=0.953), analgesic therapy with vs. without morphine (47.5±18.7 h vs. 36.9±23.0 h, respectively; p=0.107), BMI < 30 kg/m2 vs. more obese patients (40.9±21.0 h vs. 41.8±26.1 h, respectively; p=0.748), age ≥ 65 years vs. younger patients (36.3± 21.9 h vs. 45.3± 21.3 h, respectively; p=0.126), and postoperative complications (Clavien-Dindo 0: 41.4±23.1 h vs. Clavien-Dindo I: 50.4±23.8 h vs. Clavien-Dindo II: 36.0±18.4 h vs. Clavien-Dindo IIIb: 37.2±18.7 h vs. Clavien-Dindo IVa: 55.2 h; P=0.817).

**Discussion**

The postoperative status and recovery of a patient is very complex and not yet fully understood. The CTT is an objective measurement regarding gastrointestinal passage. As a result, interventions on the postoperative passage can be compared more objectively within academic research.

In 1987, Metcalf *et al.* (8) escribed the determination of radiopaque marker distribution. It is a simple, inexpensive, rapid, reproducible, readily available, and easy-to-perform examination. Anatomical differences and physiological variations in bowel movement between patients are possible influencing factors. This method represents only a snapshot of a complex physiological process. They proposed to multiply the number of the radiopaque markers remaining in the colon with a fixed factor of 2.4 to calculate the CTT. The position of the radiopaque marker in the abdominal X-ray is ignored. The groups are dichotomized into excreted versus not excreted markers. In the present study, the position of the radiopaque marker in the abdomen was differentiated between the right colon, left colon, rectosigmoid, and excreted positions. These four positions are sorted in a natural order presenting ordinal data. The use of ordinal instead of dichotomized data in combination with recent statistical developments (such as CLMMs) has enabled the authors to identify morphine use, female sex, and left-sided resection as risk factors for a less advanced marker position and a corresponding prolonged colonic transit. Given their negative impacts on the ordinal distribution of radiopaque markers in CLMMs, morphine use, female sex, and left-sided colorectal resections were identified as risk factors for postoperative ileus. The validity of CTT as a measure of postoperative ileus was confirmed by a nearly threefold higher rate of repeated postoperative vomiting in patients with a longer CTT. These results must be interpreted with care because of a potential bias caused by PONV. Its use in a regular clinical setting is restricted due to its complex and long ongoing process. Further development of this method could simplify and shorten this process as well as reduce the radiation by performing an X-ray of the stool only or by using markers that are detectable by using an external device.

The influence of avoidance of sparing of opioids on bowel function is well described and has been confirmed in the literature (9-11) providing external validation of the findings in this study. Morphine binds to the opioid receptors of the whole gastrointestinal tract and inhibits gastrointestinal motility and secretion. As a result, the passage is prolonged, and fluid resorption is increased. This can lead to opioid-induced ileus.

The slower colonic transit observed in female patients confirms previous studies conducted in the 2000s. Meier *et al.* (12) described a twelve-hour shorter CTT in men than in women. Similar results were described by Graff *et al.* (13) A more recent study by Cho *et al.* (14) described a CTT of 9 hours in male patients compared to 25 hours in female patients. These findings are in contrast to the results of Jung *et al.* (15), who failed to demonstrate a gender-specific difference in CTT in a low-powered study group. A longer CTT in female patients in the luteal phase compared to the follicular phase was identified, suggesting a possible influence of female sex hormones on CTT. Such an effect seems to be unlikely to have affected the present study results considering the median age of 63 years of the patients. A further gender difference may also be related to the higher prevalence of chronic constipation occurring in women compared to men (16).

Left-sided colorectal resection was associated with longer colonic transit than right-sided colonic resection. The number of right-sided colectomies (n=6) that occurred in this study is small. The resection of a different colon section can differentially affect colonic transit. Tomita *et al.* (17) described significantly different segmental CTT for the ascending (9.5 hours), transverse (4.2 hours), descending (5.5 hours), and rectosigmoid colon (12.7 hours). The CTT of the resected colon section determines not only the total colonic transit but also its physiological function. In right-sided hemicolectomy, resection of the ileocecal valve and the terminal ileum can result in bile acid malabsorption and small intestine bacterial overgrowth (18). Bile acid has high osmotic activity in the colon, which can cause chologenic diarrhoea. Occurring bacterial overgrowth can cause damage to the mucous membrane of the small intestine, leading to malabsorption of carbohydrates and fatty acids, resulting in diarrhoea (19, 20). The meta-analysis of Seo *et al.* (21) described prolonged postoperative ileus following right- versus left-sided colectomy. Prolonged postoperative ileus was defined as being very heterogeneous. These researchers postulated consecutive rectosigmoid hyperactivity (22). The rectosigmoid has a crucial role in defecation control and continence preservation. Patients with sigmoid or rectosigmoid resection mainly suffer from strained or obstructed defecation and incomplete evacuation due to denervation injury of the hypogastric and inferior mesenteric plexus (23).

The influence of preoperative bowel preparation on postoperative intestinal passage and ileus is discussed controversially in the current literature (4, 24, 25). All patients receiving anterior / higher rectum resection (N=4) can be found in the short CTT group. This could indicate a bias in this evaluation. However, type of surgery (*P*=0.184) and side of operation (*P*=0.962) were not significantly different between the short CTT and long CTT group.

*Limitations*

The main limitation of the present study was the secondary analysis of the data with randomization and blinding for caffeine use. Additionally, the present results were measured in patients after elective laparoscopic colorectal surgery; therefore, they are difficult to compare to results obtained in healthy probands with regular food intake.

The determination of the radiopaque marker position described by Metcalf *et al.*(8) was designed for nonoperated patients. When regarding the diagnostic value, the radiopaque marker method was defined as the gold standard (despite quite limited data in the available research) and balanced by the objectivity of this method.

**Conclusion**

Morphine use, female sex, and left-sided colorectal resection correlate with prolonged colonic transit. These parameters may be risk factors for postoperative ileus after laparoscopic colorectal resection and require more intensive laxative therapies.

**References**

1. Abbassi F, Müller SA, Steffen T, Schmied BM, Warschkow R, Beutner U, et al. Caffeine for intestinal transit after laparoscopic colectomy: randomized clinical trial (CaCo trial). The British journal of surgery. 2022;109(12):1216–23.

2. Kruse C, Müller SA, Warschkow R, Lüthi C, Brunner W, Marti L, et al. Does caffeine reduce postoperative bowel paralysis after elective laparoscopic colectomy? (CaCo trial): study protocol for a randomized controlled trial. Trials. 2016;17:186.

3. Dindo D, Demartines N, Clavien PA. Classification of surgical complications: a new proposal with evaluation in a cohort of 6336 patients and results of a survey. Annals of surgery. 2004;240(2):205–13.

4. Gustafsson UO, Scott MJ, Hubner M, Nygren J, Demartines N, Francis N, et al. Guidelines for Perioperative Care in Elective Colorectal Surgery: Enhanced Recovery After Surgery (ERAS(®)) Society Recommendations: 2018. World J Surg. 2019;43(3):659-95.

5. Agresti A, Coull BA. Approximate is better than “exact” for interval estimation of binomial proportions. The American Statistician. 1998;52(2):119–26.

6. Thiele C, Hirschfeld G. cutpointr: improved estimation and validation of optimal cutpoints in R. Journal of Statistical Software. 2021;98(11):1–27.

7. Lenz ST. Alan agresti (2013): categorical data analysis. Statistical Papers. 2016;57(3):849–50.

8. Metcalf AM, Phillips SF, Zinsmeister AR, MacCarty RL, Beart RW, Wolff BG. Simplified assessment of segmental colonic transit. Gastroenterology. 1987;92(1):40–7.

9. Gustafsson UO, Scott MJ, Hubner M, Nygren J, Demartines N, Francis N, et al. Guidelines for perioperative care in elective colorectal surgery: Enhanced recovery after surgery (ERAS(®)) society recommendations: 2018. World journal of surgery. 2019;43(3):659–95.

10. Zingg U, Miskovic D, Hamel CT, Erni L, Oertli D, Metzger U. Influence of thoracic epidural analgesia on postoperative pain relief and ileus after laparoscopic colorectal resection : benefit with epidural analgesia. Surgical endoscopy. 2009;23(2):276–82.

11. Rodriguez-Monguio R, Berkley E, Mendoza E, Miller K, Selim S, Trac C, et al. Inpatient administration of opioids and risk for post-operative ileus in older adults. Journal of opioid management. 2022;18(4):317–25.

12. Meier R, Beglinger C, Dederding JP, Meyer-Wyss B, Fumagalli M, Rowedder A, et al. Influence of age, gender, hormonal status and smoking habits on colonic transit time. Neurogastroenterology and motility : the official journal of the European Gastrointestinal Motility Society. 1995;7(4):235–8.

13. Graff J, Brinch K, Madsen JL. Gastrointestinal mean transit times in young and middle-aged healthy subjects. Clinical physiology (Oxford, England). 2001;21(2):253–9.

14. Cho KO, Jo YJ, Song BK, Oh JW, Kim YS. Colon transit time according to physical activity and characteristics in South Korean adults. World journal of gastroenterology. 2013;19(4):550–5.

15. Jung HK, Kim DY, Moon IH. Effects of gender and menstrual cycle on colonic transit time in healthy subjects. The Korean journal of internal medicine. 2003;18(3):181–6.

16. Black CJ, Ford AC. Chronic idiopathic constipation in adults: epidemiology, pathophysiology, diagnosis and clinical management. The Medical journal of Australia. 2018;209(2):86–91.

17. Tomita R, Igarashi S, Ikeda T, Sugito K, Sakurai K, Fujisaki S, et al. Study of segmental colonic transit time in healthy men. Hepato-gastroenterology. 2011;58(110-111):1519–22.

18. Larsen HM, Krogh K, Borre M, Gregersen T, Mejlby Hansen M, Arveschoug AK, et al. Chronic loose stools following right-sided hemicolectomy for colon cancer and the association with bile acid malabsorption and small intestinal bacterial overgrowth. Colorectal Dis. 2022;00:1–8.

19. Mathias JR, Clench MH. Review: pathophysiology of diarrhea caused by bacterial overgrowth of the small intestine. The American journal of the medical sciences. 1985;289(6):243–8.

20. Ghoshal UC, Sachdeva S, Ghoshal U, Misra A, Puri AS, Pratap N, et al. Asian-Pacific consensus on small intestinal bacterial overgrowth in gastrointestinal disorders: an initiative of the indian neurogastroenterology and motility association. Indian journal of gastroenterology : official journal of the Indian Society of Gastroenterology. 2022;41(5):483–507.

21. Seo SHB, Carson DA, Bhat S, Varghese C, Wells CI, Bissett IP, et al. Prolonged postoperative ileus following right- versus left-sided colectomy: a systematic review and meta-analysis. Colorectal disease : the official journal of the Association of Coloproctology of Great Britain and Ireland. 2021;23(12):3113–22.

22. Seo SHB, Bissett I, O'Grady G. Variable Gut Function Recovery After Right vs. Left Colectomy May Be Due to Rectosigmoid Hyperactivity. Front Physiol. 2021;12:635167.

23. Elfeki H, Larsen HM, Emmertsen KJ, Christensen P, Youssef M, Khafagy W, et al. Bowel dysfunction after sigmoid resection for cancer and its impact on quality of life. The British journal of surgery. 2019;106(6):805.

24. Woodfield JC, Clifford K, Schmidt B, Turner GA, Amer MA, McCall JL. Strategies for Antibiotic Administration for Bowel Preparation Among Patients Undergoing Elective Colorectal Surgery: A Network Meta-analysis. JAMA Surg. 2022;157(1):34-41.

25. Kiran RP, Murray AC, Chiuzan C, Estrada D, Forde K. Combined preoperative mechanical bowel preparation with oral antibiotics significantly reduces surgical site infection, anastomotic leak, and ileus after colorectal surgery. Ann Surg. 2015;262(3):416-25; discussion 23-5.

**Table S1**

Radiopaque marker indigested on postoperative day (POD) 1, 2, and 3 and 1-3. The position was obtained from an abdominal X-ray on POD 4.

| **Localization on POD 4** | **POD of Indigestion** | **Total** | **Left-sided resection** | **Right-sided resection** | **Male** | **Female** | **No morphine** | **Morphine** |
| --- | --- | --- | --- | --- | --- | --- | --- | --- |
| Small bowel | 3 | 10 (0.7%) | 10 (0.7%) | 0 (0.0%) | 10 (1.1%) | 0 (0.0%) | 10 (1.1%) | 0 (0.0%) |
| Right colon | 1 | 168 (11.0%) | 157 (11.6%) | 11 (6.1%) | 103 (11.4%) | 65 (10.3%) | 85 (9.1%) | 83 (13.8%) |
|  | 2 | 186 (12.2%) | 163 (12.1%) | 23 (12.8%) | 93 (10.3%) | 93 (14.8%) | 99 (10.6%) | 87 (14.5%) |
|  | 3 | 200 (13.1%) | 186 (13.8%) | 14 (7.8%) | 118 (13.1%) | 82 (13.0%) | 100 (10.8%) | 100 (16.7%) |
| Left colon | 1 | 50 (3.3%) | 42 (3.1%) | 8 (4.4%) | 25 (2.8%) | 25 (4.0%) | 26 (2.8%) | 24 (4.0%) |
|  | 2 | 52 (3.4%) | 49 (3.6%) | 3 (1.7%) | 23 (2.6%) | 29 (4.6%) | 24 (2.6%) | 28 (4.7%) |
|  | 3 | 83 (5.4%) | 63 (4.7%) | 20 (11.1%) | 45 (5.0%) | 38 (6.0%) | 55 (5.9%) | 28 (4.7%) |
| Rectosigmoid | 1 | 39 (2.5%) | 29 (2.1%) | 10 (5.6%) | 20 (2.2%) | 19 (3.0%) | 26 (2.8%) | 13 (2.2%) |
|  | 2 | 32 (2.1%) | 27 (2.0%) | 5 (2.8%) | 16 (1.8%) | 16 (2.5%) | 22 (2.4%) | 10 (1.7%) |
|  | 3 | 53 (3.5%) | 45 (3.3%) | 8 (4.4%) | 30 (3.3%) | 23 (3.7%) | 30 (3.2%) | 23 (3.8%) |
| Excreted | 1 | 253 (49.6%) | 222 (16.4%) | 31 (17.2%) | 152 (16.9%) | 101 (16.0%) | 173 (18.6%) | 80 (13.3%) |
|  | 2 | 240 (47%) | 211 (15.6%) | 29 (16.1%) | 168 (18.7%) | 72 (11.4%) | 165 (17.7%) | 75 (12.5%) |
|  | 3 | 164 (32.2%) | 146 (10.8%) | 18 (10.0%) | 97 (10.8%) | 67 (10.6%) | 115 (12.4%) | 49 (8.2%) |

**Table S2**

Uni- and multivariable mixed effects cumulative link model for ordinal distribution of radiopaque markers on X-ray.

| **Variable** | **Label** | **n** | **Univariable**  **OR (95% CI)** | ***P* value** | **Multivariable**  **OR (95% CI)** | ***P* value** | |
| --- | --- | --- | --- | --- | --- | --- | --- |
| Caffeine | No | 17 | Ref | **0.005** | Ref | 0.060 | |
|  | Any | 34 | 1.42 (1.11 to 1.82) |  | 1.29 (0.99 to 1.69) |  | |
| Morphine | No | 31 | Reference | **<0.001** | Reference | **<0.001** | |
|  | Any | 20 | 0.38 (0.29 to 0.51) |  | 0.44 (0.32 to 0.59) |  | |
| Sex | Male | 30 | Reference | **<0.001** | Reference | **<0.001** | |
|  | Female | 21 | 0.47 (0.36 to 0.62) |  | 0.44 (0.33 to 0.60) |  | |
| Age (years) | < 65 | 27 | Reference | **0.004** | Reference | 0.106 | |
|  | ≥ 65 | 24 | 1.44 (1.13 to 1.85) |  | 1.25 (0.95 to 1.65) |  | |
| BMI (kg/m^2) | < 30 | 41 | Reference | 0.620 | Reference | 0.839 | |
|  | ≥ 30 | 10 | 0.92 (0.67 to 1.26) |  | 1.04 (0.72 to 1.50) |  | |
| Side of operation | Left | 45 | Reference | 0.443 | Reference | **0.006** | |
|  | Right | 6 | 1.17 (0.78 to 1.75) |  | 2.02 (1.21 to 3.38) |  | |
| OR: Odds ratio; 95% CI: 95% confidence interval | | | | | | |  |

**Figure S1**

Abdominal X-ray imaging (posterior-anterior) with division of the large intestine in the right colon (R), left colon (L), and rectosigmoid (RS) according to the method described by Metcalf et al. (8)


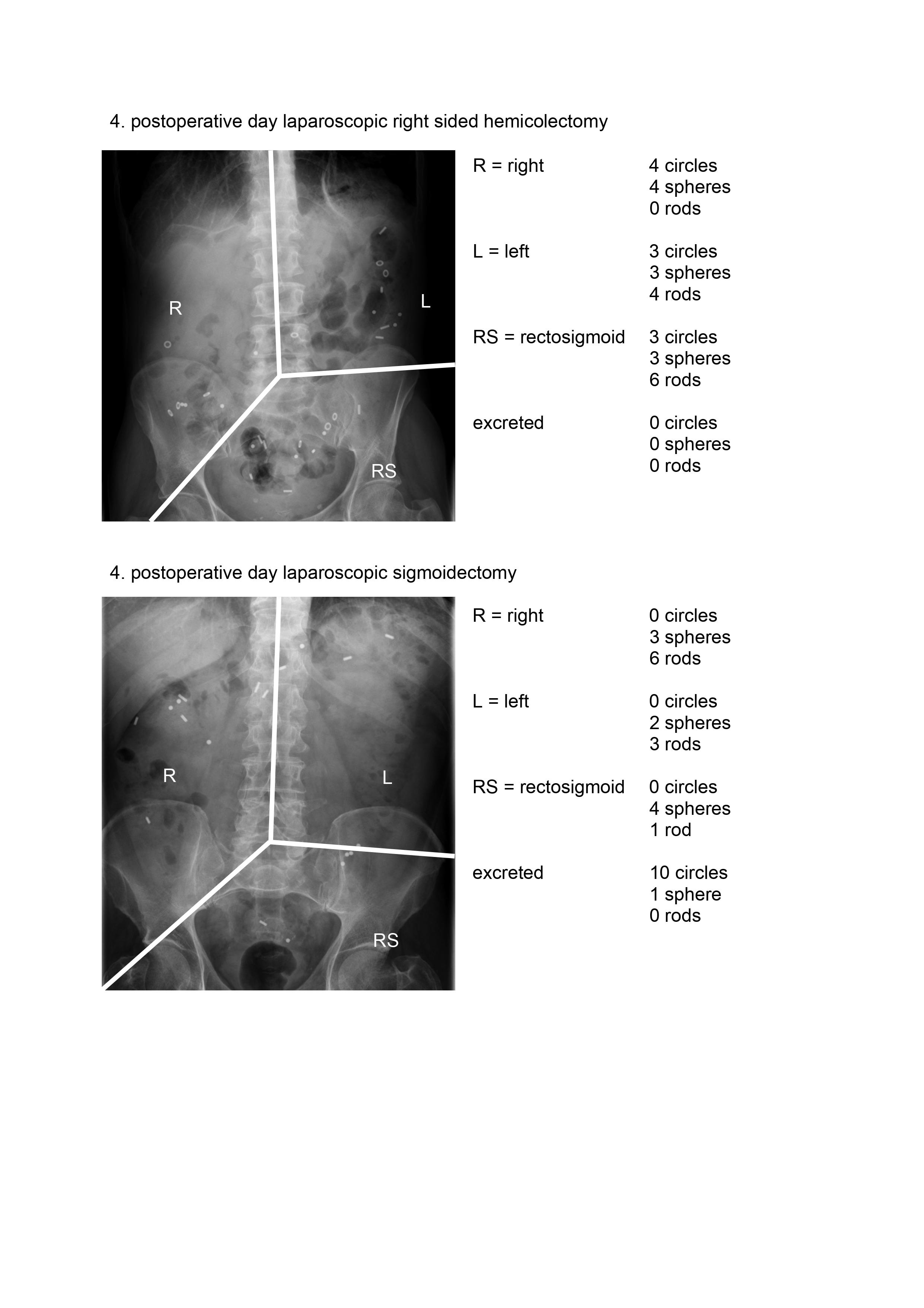

Supplement: zrad111_Supplementary_Data [file zrad111_supplementary_data.docx]
